# P-958. Leveling Up Education Interventions: Antimicrobial Stewardship and Infection Prevention Education Through Gamification

**DOI:** 10.1093/ofid/ofae631.1148

**Published:** 2025-01-29

**Authors:** Nicole M Hall, Haodi Ruan, Mary duncan, Rachael A Lee, Jeremey Walker

**Affiliations:** UAB Hospital, Birmingham, Alabama; UAB, Birmingham, Alabama; University of Alabama-Birmingham, birmingham, Alabama; University of Alabama at Birmingham, Birmingham, AL; University of Alabama Birmingham, Birmingham, Alabama

## Abstract

**Background:**

Kaizen-Education is an app-based, formative question bank infused with gamification for a fun, yet competitive learning environment. This provides an engaging and flexible way to learn new competencies and test retention. Originally developed at University of Alabama at Birmingham (UAB), it has been used in multiple areas of medical education with high learner engagement. Optional participation, team-based competition, leaderboards, and prizes are game elements used to motivate completion of the formative questions. We created two educational games, one for UAB Antimicrobial Stewardship Program (ASP) and another for Infection Prevention (IP), to see if we could engage learners within the hospital system surrounding these topics.

**Figure:**
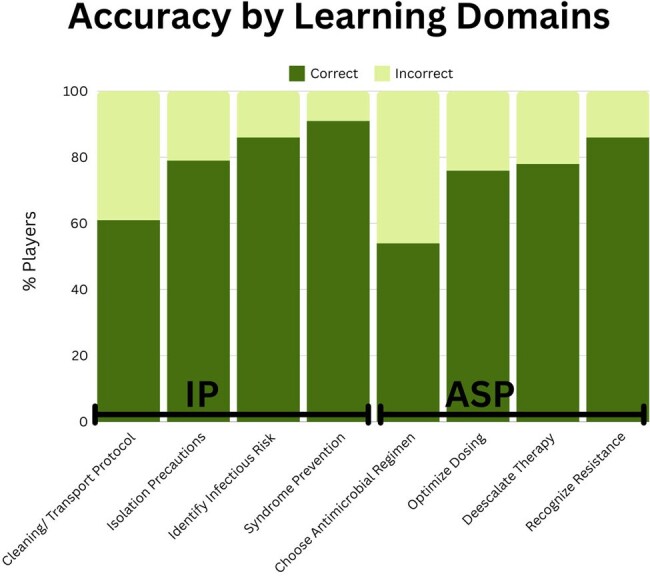

**Methods:**

For the IP game, infection preventionists developed questions for nursing staff. For the ASP game, ID pharmacists developed questions for providers. Both occurred within special weeks: National Nurses Week and U.S. Antibiotic Awareness Week. Recruitment strategies for both games included email invitations and peer-to-peer communication.

Teams for the IP game were nursing units, while the ASP game allowed creation of teams of 4 providers. Winning teams were awarded prizes and were recognized via email, certificate, and highlighted on the institution's website. At the conclusion of the games, participants were invited to provide feedback through a post-game survey.

Participant Survey Results

**Figure:**
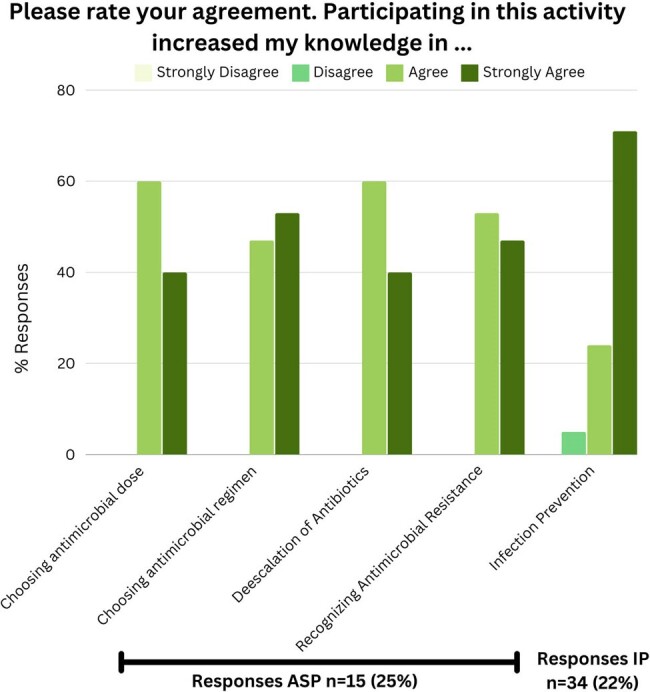

**Results:**

61 and 156 participants answered at least one question in the ASP and IP games respectively. Retention, defined as completing all questions, was 80% of players in ASP game and 69% in IP game. Across both games, 4,182 questions were answered representing unique teaching moments. Reviewing accuracy based of learning domains (Figure 1), we were able to identify knowledge gaps at our institution. Finally almost all learners who responded to feedback survey agreed the activity increased knowledge in relevant domains (Figure 2).

**Conclusion:**

We found a group of both nurses and providers interested in participating in quality educational activities within Antibiotic Stewardship and Infection Prevention. Retention was high in those that began the activity. Finally, feedback from question performance could be used to identify knowledge gaps for future interventions.

**Disclosures:**

**All Authors**: No reported disclosures

